# Model construction and application for predicting pre-eclampsia by Sonoclot coagulation analyzer

**DOI:** 10.1016/j.ncrna.2024.02.008

**Published:** 2024-02-10

**Authors:** Hongyu Shi, Weijie Wang, Fan Li, Ao Guo, Tiecheng Liu

**Affiliations:** a Endoscopic Center of the First Hospital of Jilin University, China; b Department of Gastroenterology, First Hospital of Jilin University, China; c Anesthesiology Department of Jilin University Second Hospital, Changchun, China

**Keywords:** Preeclampsia, Clot rate, Platelet function, Activated clotting time, Platelet count

## Abstract

Maternal age has significantly increased among Chinese women, thereby posing risk of pregnancy-related complications. Preeclampsia is a leading cause of maternal and perinatal morbidity and mortality, and coagulation analysis in conjunction with clinical signs and symptoms are generally used for its diagnosis with limited efficacy. Sonoclot coagulation analyzer is effective in assessing coagulation function used during cerebral surgery and cardiovascular surgery. However, its use has not been explored in preeclampsia. Here, we investigated the potential use of Sonoclot in diagnosing preeclampsia in obstetrics cases. Subjects meeting the screening criteria were divided either into a test group or a control group, according to whether they were preeclamptic or not. We recorded the Sonoclot-derived coagulation and the routine coagulation parameters including platelet function (PF), activated clotting time (ACT) and clot rate (CR), prothrombin time (PT), activated partial thromboplastin time (APTT), thrombin time (TT), fibrinogen (FIB), and platelet count. Regression analysis was done on the relevant parameters to assess the feasibility of Sonoclot analyzer in preeclampsia diagnosis. In parallel, changes in preeclampsia lncRNAs was also evaluated. Significant differences were recorded in PT and ACT between the two groups. In the monovariant logistic regression, PT and ACT appeared to be reliable predictor variables. In the multinomial logistic regression, a total of five regression steps were performed with decreasing AIC values. The K-fold cross validation resulted in an accuracy rate (ACC) of 77.5%, a false positive rate of 16.4%, and a false negative rate of 33.2%. lncRNAs ANRIL and HOXD-AS1 were found deregulated. Our findings indicate that Sonoclot may be useful for diagnosis of preeclampsia in obstetrics.

## 1 Introduction

The increased maternal age in recent years has led to an increase in the incidence of pregnancy complications. Preeclampsia (PE) may cause placental abruption, acute renal failure, intracranial hemorrhage, and in severe cases, can potentially endanger the life of women in labor [1]. The pathogenesis of preeclampsia has not yet been fully elucidated, and most researchers favor a two-stage theory to explain it. The first stage is due to inadequate recasting of the spiral arteries caused by superficial trophoblast implantation, while the second stage is due to maternal endothelial cell dysfunction and imbalance between angiogenic and anti-angiogenic factors [2]. According to the National Institute for Health and Clinical Excellence (NICE), a woman should be considered as having a high risk of PE if she has any one of the following conditions: diabetes, autoimmune diseases, chronic hypertension, chronic kidney disease, or prior hypertensive disorders in pregnancy. In addition, a woman may be deemed to be high risk if she meets two of the following criteria: first birth age under 40, body mass index below 35 kg/m^2^, a family history of PE, or a gestational age between pregnancies of at least ten years [3]. The NICE guidelines for screening, however, have a low detection rate, are ineffective at predicting the likelihood of PE, and have a high proportion of false positives. For example, while using the NICE guidelines for screening, the detection rate for simple PE, full-term PE, and false positives was 39%, 34%, and 10.3%, respectively [4]. This could result in underdiagnosis of high-risk mothers on one hand and to overmedication on the other, and hence there is a need for developing a novel diagnostic method.

The Sonoclot analyzer determines various parameters related to coagulation including fibrinogen monomer formation, and fibrinolysis in whole blood, plasma, or citrate-containing blood samples [5]. The analyzer can also evaluate values including sonoclot-activated clotting time (SonAct), platelet function (PF), clotting rate (CR) and time to peak (TP). SonACT is similar to the conventional activated clotting time (ACT), which is mainly related to clotting factors. PF and CR respectively indicate platelet function independent of platelet number and plasma fibrinogen level. TP indicates the speed of clotting contraction and is an indirect measure of the strength of platelet function. The Sonoclot analyzer has been traditionally employed in liver transplantat surgery, cardiovascular surgery, neurosurgery and other surgeries involving significant bleeding volume. Lately, it is also used in intensive care of pregnant women and children [[6], [7], [8], [9]]. Here, we investigated the feasibility of applying Sonoclot in the diagnosis of PE.

## 2 Methods

This single-center study was approved by the Research Ethics Committee of the First Hospital of Jilin University. All patients included in the study signed the relevant informed consent forms. The study population was patients who visited the obstetrics department from March 2021 to August 2022. Patients included in the study were required to meet the following criteria: (1) fasting, (2) no history of alcohol consumption in the last 3 months, (3) no history of drug use, (4) no history of lipid-lowering medication, (5) good rest in the last 3 months, and (6) negative maternal urine protein test results in the normal group. Exclusion criteria: (1) those with cardiovascular disease, diabetes, hyperthyroidism, and rheumatic diseases; (2) those with combined tuberculosis, chronic kidney disease and chronic hepatitis; (3) those with twin fetuses and fetal malformations; and (4) those with severe immunological diseases, malignant tumors and inflammatory diseases.

By these criteria, a total of 121 patients were included in the study, and all patients were divided into an experimental group (77 patients) and a control group (44 patients) according to the presence or absence of typical preeclampsia symptoms. All pregnant women in the preeclampsia group met the relevant diagnostic criteria for preeclampsia. After admission, 3 ml venous blood was drawn and discarded, and then 2 ml of venous blood was drawn as a sample for subsequent use.

### 2.1 Operation procedure of sonoclot analyzer

The Sonoclot coagulation analyzer was preheated to 37 °C, and 1 ml venous blood was drawn and pushed out into the blood cup cup that was placed on a reaction cell that could be rotated back and forth at an angle of 4°45'. The data generated by the analyzer was collected and divided into two predesignated groups, and presented as mean ± SD. For the test of significance, we used Student's t-test. A visual violin plot was also drawn based on the obtained data.

### 2.2 Model construction

Logistic regression applicable to binary response variables were selected for this study and the final results were based on the multivariate logistic regression results. The univariate logistic regression results were used as a reference for screening the predictor variables. In the process of identifying predictor variables, we calculated the Akaike Information Criterion (AIC) value for each model, and in the case of decreasing AIC, we concluded that the newly constructed model better reflects the true situation. We also calculated the overdispersion for each model. When the overdispersion test was significant (p < 0.05), it meant that the variance of the observed response variable was larger than the variance of the expected binomial distribution, wherein we applied the logistic regression of the negative binomial distribution model.

### 2.3 Model evaluation

After constructing the prediction model, we first analyzed its multicollinearity and calculated the variance inflation factor (VIF) of the fitted model. We determined that VIF> 4 indicated the possibility of multicollinearity. Where this happened, we applied a elastic net approach to attenuate their mutual multicollinearity by adding a penalty to the coefficients in the fitted regression model. Finally, to prevent our model from underfitting or overfitting, we evaluated the performance of the model by applying the K-fold cross-validation method.

### 2.4 LncRNA evaluation

RNA was extracted using Direct-zol RNA Miniprep extraction kit (Zymo research, USA) and we detected the levels of lncRNAs by emplying quantitative real-time PCR (qPCR) using QuantiNova SYBR Green PCR kit (Qiagen, China), following the instructions from the manufacturer, and as described recently [10].

### 2.5 Statistical evaluation

T-test was used to compare the findings from two groups, experimental vs. control, under the guidance of a statistician. p values less than 0.05 were considered significant.

## 3 Results

**Fig. 1:**
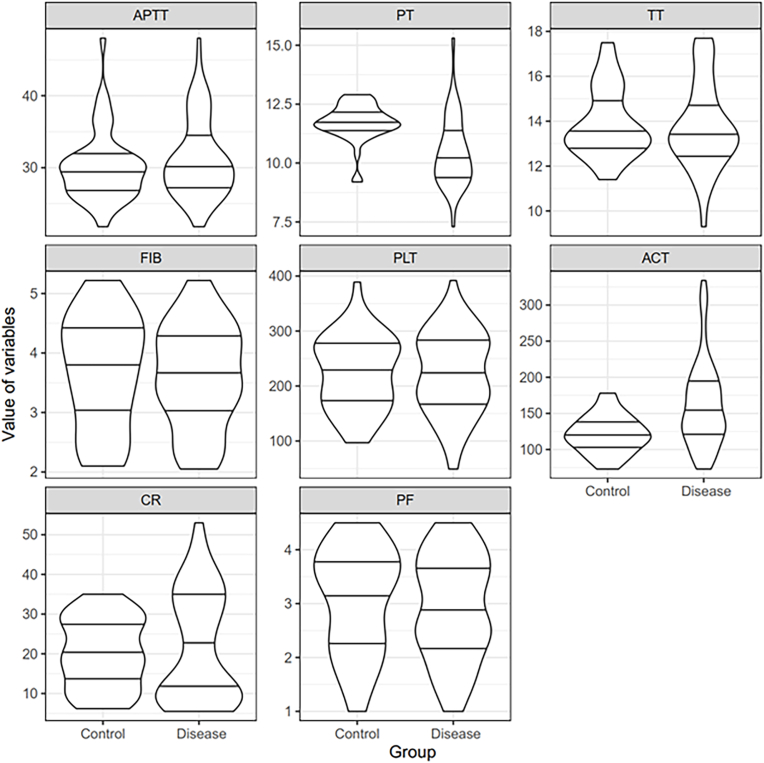
Violin plot of the indicators for the disease group and the control group.

The data distribution are presented in Table 1 and Fig. 1. The data from both groups were analyzed by t-test which showed the prolongation of PT and the change in the ACT values between the groups to be statistically significant, thus suggesting that PT and ACT were reliable predictor variables (Table 1). The results of monovariant logistic regression are shown in Table 2, which confirmed that PT and ACT were reliable predictor variables. In the multivariate logistic regression (Table 3 and Table 4), a total of five regression steps were performed with decreasing AIC values. The model fitted at each step showed no evidence of overdispersion (p > 0.05). There was no evidence of multicollinearity in the models fitted at each step (VIF <4). The K-fold method of cross-validation resulted in an accuracy rate (ACC) of 77.5%, a false positive rate (FPR) of 16.4%, and a false inverse rate of 33.2%.

**Table 1:** *t*-test results.

Variables	Mean of control	SD of control	Mean of disease	SD of disease	T value	Degree of freedom	P Value
APTT	30.11395349	4.881577682	31.19210526	5.512646386	−1.103815678	96.37647759	0.272421505
PT	11.72325581	0.784625239	10.47539474	1.488152717	5.98602361	116.5587118	2.43 × 10^−8^
TT	13.90697674	1.543403066	13.76447368	1.9244188	0.441615162	103.5274572	0.659688739
FIB	3.689767442	0.946327459	3.611052632	0.866443426	0.449217248	81.07572882	0.654473492
PLT	224.3488372	67.48681837	222.7631579	75.31743816	0.118006485	95.49547931	0.906310378
ACT	121.1395349	26.02524543	161.9868421	59.44285624	−5.177423743	111.5613287	1.01 × 10^−6^
CR	20.12325581	8.727538168	21.79605263	13.76762813	−0.809955649	115.4094193	0.419632568
PF	3.06744186	0.994681315	2.921052632	0.969441872	0.778323628	85.45405129	0.438528052

SD: standard deviation.

**Table 2:** Results of monovariant logistic regression.

Variables	OR(95%CI)	Beta	P value	P value of OD
APTT	1.04(0.97–1.12)	−0.669018117	0.287258441	0.430792632
PT	0.46(0.32–0.66)*	9.274075385	2.52 × 10^−5^	0.232058365
TT	0.96(0.78–1.18)	1.18762968	0.675577584	0.432316713
FIB	0.9(0.59–1.38)	0.934135015	0.642931262	0.430496899
PLT	1(0.99–1)	0.637952122	0.90811663	0.43115687
ACT	1.02(1.01–1.03)*	−2.48686264	0.000202033	0.718022099
CR	1.01(0.98–1.04)	0.328139242	0.47039984	0.437399847
PF	0.86(0.58–1.26)	1.036734333	0.431254131	0.430223634

CI: confidence interval; OD: overdispersion; * refer to significant results.

**Table 3:** Results of multivariable logistic regression.

Variables	Beta	SE	P Value	OR	Upper of 95% CI	Lower of 95% CI
(Intercept)	2.182441058	2.763978326	0.429760611	8.867926985	1997.873365	0.039361919
APTT	0.091322436	0.049646345	0.065847642	1.095622217	1.20759318	0.994033472
PT	−0.866239476	0.210002155	3.71 × 10^−5^	0.420529993	0.634679424	0.278637479
ACT	0.031651849	0.008274395	0.000130625	1.032158096	1.049033919	1.015553755
CR	0.042693481	0.022730207	0.060344145	1.043617957	1.091163639	0.998144

CI: confidence interval; SE: standard error; OR: odds ratio.

**Table 4:** Model selection process based on AIC values.

Steps	Formula	AIC	P value of OD	VIF value	Advices
1	mrk ∼ APTT + PT + TT + FIB + PLT + ACT + CR + PF	120	0.557383816	1.397281936	delete PF
2	mrk ∼ APTT + PT + TT + FIB + PLT + ACT + CR	118	0.497255629	1.350604228	delete PLT
3	mrk ∼ APTT + PT + TT + FIB + ACT + CR	116	0.520776154	1.345347337	delete FIB
4	mrk ∼ APTT + PT + TT + ACT + CR	114	0.533914084	1.33968588	delete TT
5	mrk ∼ APTT + PT + ACT + CR	113	0.467691737	1.223057291	best fitted

AIC: Akaike information criterion; OD: overdispersion.

**Fig. 2:**
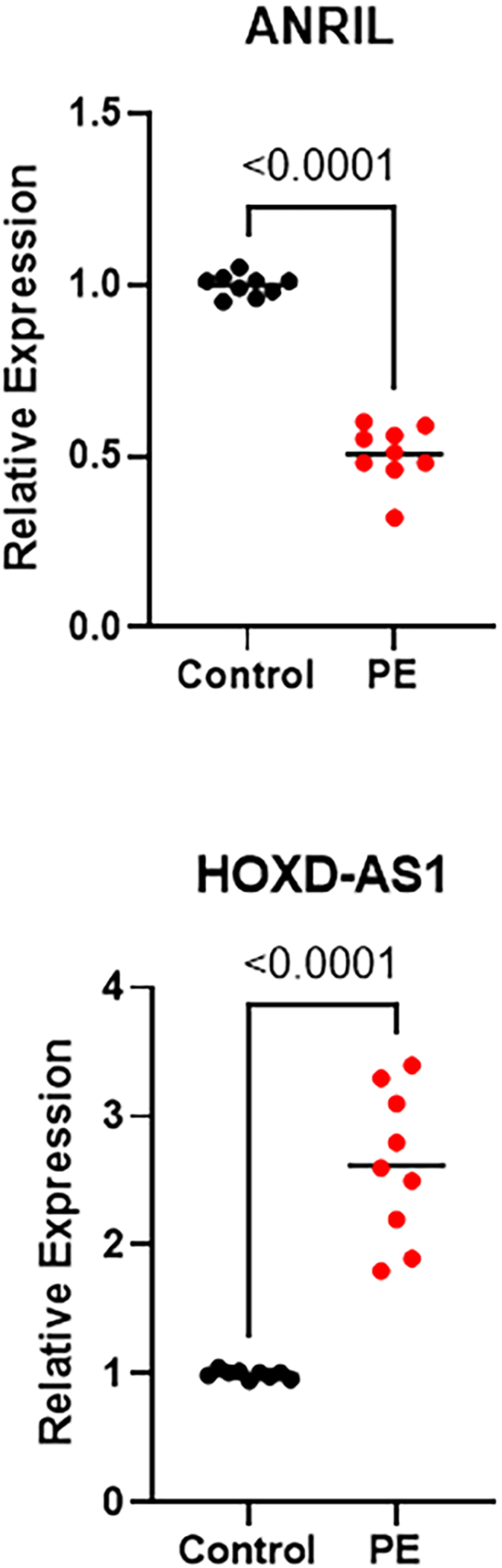
Levels of lncRNAs ANRIL and HOXD-AS1 in preeclampsia (PE) patient, compared to controls. Levels of lncRNAs were detected by quantitative real time PCR and absolute values were compared. p values of relative expression are provided.

Additionally, we also sought to study a possible deregulation of non-coding RNAs such as ANRIL and HOXD-AS1 in preeclampsia. Our results show that the levels of these two lncRNAs show different trends in preeclampsia patients. ANRIL was significantly reduced and downregulated to less than half the levels in preeclampsia patients, when compared to control group (p < 0.001). At the same time, HOXD-AS1 was significantly upregulated in preeclampsia patients, when compared to control group, p < 0.001 (Fig. 2).

## 4 Discussion

Preeclampsia is one of the most common obstetric disorders, with approximately 40,000 maternal deaths per year due to this disorder during pregnancy. In a cross-sectional study, it was revealed that the incidence in China is quote similar to western countries but whereas two-thirds of cases in Sweden are mild, the corresponding two-thirds of cases in China are severe [11]. The pathogenesis is not yet fully elucidated and the diagnosis of preeclampsia is complex [12]. The diagnosis is often made by combining proteinuria and hypertension, although this approach has limited use because both symptoms are secondary, downstream signs of the disease. More recent critera also include maternal organ dysfunction such as renal insufficiency, liver involvement, neurological or hematologic complications, subplacental dysfunction, or fetal growth restriction [13]. If left untreated, preeclampsia can be fatal, and the condition is one of the leading causes of maternal and child mortality in under-resourced settings [14]. Research is underway to improve the diagnosis of preeclampsia and to enable universal screening. As described before, NICE guideline has limited use in determining the risk factor for developing PE in pregnant women [4].

PLGF is a glycosylated dimeric protein secreted by trophoblast cells and is a member of the vascular endothelial growth factor family. According to studies, lower PLGF levels may be associated with the development of PE in compared with women with normal pregnancy [[15], [16], [17]]. By assessing the ratio of soluble fms-like tyrosine kinase 1 (sFlt-1) to placental growth factor (PlGF) in pregnant women prior to clinical PE inferred that an sFlt-1:PlGF ratio of 38 or lower can be used to predict the temporary absence of PE in whom the condition is clinically suspected [18]. Low serum pregnancy associated plasma protein A (PAPP-A), which is a key regulator of insulin-like growth factor bioavailability and is essential for normal fetal development is associated with adverse pregnancy outcome, albeit with a poor predictive value [19]. Abnormal placental activity that characterizes PE is associated with increased vascular resistance in the placenta, and could be diagnosed by ultrasonographic evidence such as an increase in the pulsatility index (PI) of the uterine artery or persistent diastolic ‘notch’ in its Doppler waveform. The International Society of Ultrasound in Obstetrics and Gynecology (ISUOG) recently published practice guidelines on the role of ultrasound in the screening and follow-up of PE [20].

Damaged vascular endothelium and imbalances in the three major systems including the coagulation, anticoagulation, and fibrinolysis are currently thought to be related to the incidence of PE [18,21,22]. Several studies [[23], [24], [25]] have shown that coagulation-related indicators alter in PE-affected women, however conventional coagulation tests only provide data on a specific, discrete element of the coagulation process, which is insufficient for comprehending the entire coagulation process. By contrast, the Sonoclot coagulation analyzer can rapidly and conveniently monitor the entire coagulation process in a small amount of serum [5]. In this study, the traditional coagulation indexes and the measurable values of Sonoclot coagulation analyzer were recorded in two groups of mothers. The *t*-test was first performed to assess the concordance of the data between the two groups. The analyses suggested that patients who developed PE had significantly shorter PT and longer ACT. These findings are consistent with the patient being in a hypercoagulable state to maintain the integrity of the placenta. Persistent hypercoagulable state, secondary to overactivation of the fibrinolytic system, causes a prethrombotic state, which eventually develops into PE.

When all indicators of the study were included in a multivariate logistic regression with cross-validation, we observed a model accuracy (ACC) of 77.5% and a false positive rate of 16.4%. According to the 2019 screening guidelines of the International Federation of Obstetrics and Gynecology (IFOG), the combination of PlGF, PAPP-A, MAP, and UtPI is the most accurate predictor of PE [26]. In a large prospective study conducted by Akolekar R et al. at the U.K.’s Fetal Medicine Foundation (FMF), the detection rate of PE in pregnant women was 96% when combined PAPP-A, PlGF, UtPI, and MAP was applied with a false positive rate of 10% [27]. An Asian multicenter prospective study reported that the combined early pregnancy multiplex screening approach had a 71.8% detection rate for PE with a false-positive rate of 15% [26]. A prospective Chinese multicenter study reported that the detection rate of PE by combined multiplex screening in early pregnancy was 72.6 % with a false-positive rate of 15% [28]. These studies along with our present findings underscore that Sonoclot is an useful guide for the early diagnosis of PE, and in combination with traditional coagulation-related indicators it can significantly improve the detection rate of PE.

In recent years, non-coding RNAs have attracted a lot of attention in PE research [[29], [30], [31]] even though these same non-coding RNAs were considered junk not very long ago [32]. In our patient population, we checked the expression of two specific lncRNAs, ANRIL and HOXD-AS1. The two lncRNAs were specifically chosen because of their demonstrated role in PE. ANRIL's role [33,34] as well as HOXD-AS1's role [35,36] in PE has been published before and our own results are in agreement with the earlier findings.

Despite all the promoses, our study also has some limitations including small sample size and a single-center study, which could somewhat affect the accuracy of the results.

## Funding

This research was funded by the Project of Science and 10.13039/100006180Technology Development Plan of 10.13039/501100003807Jilin Province (20190304116YY).

## CRediT authorship contribution statement

**Hongyu Shi:** Writing – original draft, Formal analysis. **Weijie Wang:** Writing – review & editing, Data curation. **Fan Li:** Writing – review & editing, Formal analysis. **Ao Guo:** Validation, Methodology, Investigation. **Tiecheng Liu:** Writing – review & editing, Supervision, Software, Resources, Conceptualization.

## Declaration of competing interest

The authors declare that they have no known competing financial interests or personal relationships that could have appeared to influence the work reported in this paper.
